# Haemodynamics from Induction to Cardiopulmonary Bypass Improve Prediction of Right Ventricular Failure Following Left Ventricular Assist Device

**DOI:** 10.1093/icvts/ivag215

**Published:** 2026-07-29

**Authors:** Shruti Rajendra, Yousuf Salmasi, Binu Raj, Owais Dar, Nandor Marczin, Darrel Francis, Espeed Khoshbin

**Affiliations:** Faculty of Medicine, Imperial College London, London, United Kingdom; Faculty of Medicine, Imperial College London, London, United Kingdom; Cardiac Surgery, Royal Brompton and Harefield Hospitals, London, United Kingdom; Harefield Hospital, Royal Brompton and Harefield Hospitals, London, United Kingdom; Cardiology, Harefield Hospital, Royal Brompton and Harefield Hospitals, London, United Kingdom; Faculty of Medicine, Kings College, London, United Kingdom; Faculty of Medicine, Imperial College London, London, United Kingdom; Department of Anaesthesia and Intensive Care, Harefield Hospital, London, United Kingdom; Faculty of Medicine, Imperial College London, London, United Kingdom; Cardiology, Hammersmith Hospital, Imperial College London, London, United Kingdom; Faculty of Medicine, Imperial College London, London, United Kingdom; Cardiac Surgery, Royal Brompton and Harefield Hospitals, London, United Kingdom

**Keywords:** left ventricular assist device, right ventricular failure, prediction

## Abstract

**Objectives:**

Right ventricular failure (RVF) remains a frequent complication following left ventricular assist device (LVAD) implantation. Conventional risk scores rely on preoperative data acquired under stable conditions, which may overlook early signs of RV decompensation that emerge under perioperative stress. This study evaluates whether perioperative haemodynamic markers prior to cardiopulmonary bypass (CPB) improve RVF prediction compared to preoperative assessment obtained by right heart catheterization (RHC), echocardiographic parameters, and scoring systems such as the European Registry for Patients with Mechanical Circulatory Support (EUROMACS) RHF score.

**Methods:**

We conducted a retrospective single-centre study of 203 LVAD patients (2013-2023). The primary outcome was RVF. Preoperative data included clinical variables, echocardiography, and RHC. Perioperative data included vasoactive-inotropic score (VIS), pulmonary artery (PA) catheter, and arterial-line monitoring between induction and start of CPB. Two multivariable models were developed using least absolute shrinkage-and-selection operator (LASSO) logistic regression: (1) Preoperative model; (2) Pre-CPB model. Model performance was assessed using area under the curve (AUC) and compared against EUROMACS-RHF using DeLong tests.

**Results:**

Thirty-six patients (17.7%) developed RVF. No preoperative RHC-derived parameter independently predicted RVF. The Preoperative model achieved moderate discrimination (AUC: 0.747). Incorporating intraoperative mean arterial pressure (MAP), pulmonary artery pulsatility index (PAPi), and maximum VIS yielded a Pre-CPB model with strong discrimination (AUC: 0.834). The Pre-CPB model significantly outperformed both EUROMACS-RHF (*P* = 0.0299) and the Preoperative model (*P* = 0.049), with no significant difference between our Preoperative model and EUROMACS-RHF (*P* = 0.222).

**Conclusions:**

Perioperative markers prior to CPB significantly outperform preoperative prediction of RVF post-LVAD. Incorporating these into real-time risk stratification can improve decision-making, enabling earlier RV support in the operating theatre for high-risk LVAD patients.

## Graphical abstract

**Figure:**
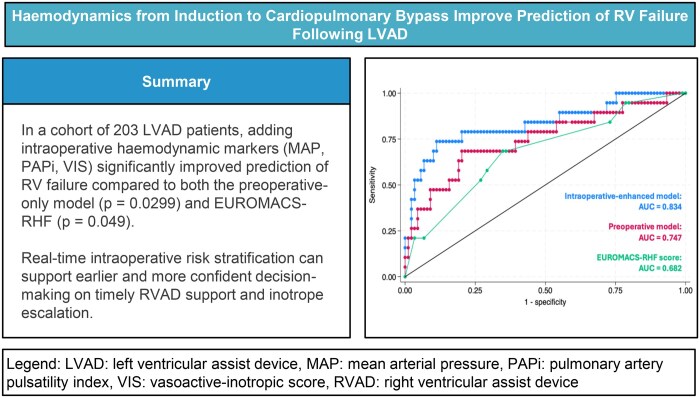

## INTRODUCTION

Right ventricular failure (RVF) is a serious complication following left ventricular assist device (LVAD) implantation, affecting approximately 20%-40% of patients.^1–3^ Right ventricular failure contributes to prolonged intensive therapy unit (ITU) stays and significantly increased short and long-term morbidity and mortality.^1–3^ Accurate identification of high-risk patients is critical to facilitate timely RV support. Early temporary right ventricular assist device (RVAD) implantation has been shown to improve outcomes, while delayed RVAD placement is associated with higher mortality.^4^^,^^5^

Despite this clinical burden, reliable prediction of RVF remains challenging.^6^ Several preoperative RVF risk prediction scores—including the EUROMACS-RHF, right ventricular failure risk score (RVFRS), and the Drakos score—have been developed using clinical characteristics, echocardiographic findings, and right heart catheterization (RHC) values.^7–9^ However, their predictive performance remains modest, with similar or poorer performance in external validation.^5–9^ These models rely on preoperative measurements acquired under stable conditions, typically days or weeks prior to LVAD surgery. This does not account for the acute haemodynamic stressors encountered during the perioperative period.^7–9^ During LVAD implantation, the RV is exposed to several acute stressors, including general anaesthesia, cardiopulmonary bypass (CPB), surgical manipulation, and fluid shifts, which can alter RV preload, pulmonary vascular resistance or afterload and RV contractility.^10^ These abrupt changes may unmask subclinical RV dysfunction that is not evident preoperatively, highlighting the perioperative period as a potentially critical window for risk stratification.

Several studies have demonstrated the prognostic value of postoperative haemodynamic markers—including central venous pressure (CVP), diastolic pulmonary artery pressure (dPAP), pulmonary artery pulsatility index [PAPi = (systolic pulmonary artery pressure (sPAP) − dPAP)/CVP], and mean arterial pressure (MAP)—for predicting RVF.^11–14^ One study incorporating CVP and dPAP measured immediately after chest closure reported an area under the curve (AUC) of 0.78, slightly exceeding existing preoperative risk scores.^14^ However, these predictions are made after chest closure, by which time the opportunity for preemptive RVAD insertion without resternotomy has already passed. Recent evidence highlights the value of intraoperative haemodynamic assessment for RVF prediction.^5^^,^^13^ For example, PAPi measured prior to CPB weaning—and to a lesser extent, CVP—have been identified as significant predictors of RVF.^13^

Nevertheless, it is not known whether a risk model based on haemodynamic values in the pre-CPB timeframe improves prediction. This study: (1) develops a novel perioperative prediction model based on haemodynamic parameters between anaesthetic induction and the start of CPB, and (2) evaluates whether this improves prediction of RVF following LVAD, in comparison to preoperative assessment and established preoperative risk scores.

## METHODS

### Study design and patient selection

We conducted a single-centre retrospective observational study including all patients who underwent LVAD implantation with magnetically levitated devices (HeartWare and HeartMate III) in our cohort between March 2013 and March 2023. Patients were excluded if they underwent pump exchange, total artificial heart (TAH), non-HeartWare/HeartMate III LVAD implantations, or concomitant RVAD insertion during the LVAD surgery. Two hundred and eleven patients met the above criteria, of whom 8 were excluded due to incomplete data (1 for insufficient perioperative data and 7 for inadequate follow-up data to determine postoperative RVF status). The final study cohort therefore consisted of 203 patients.

The primary outcome measure was early post-implant RVF, defined as the need for RVAD implantation, or the presence of persistent RV dysfunction (CVP >18 mmHg and cardiac index <2.0 L/min/m^2^ in the absence of elevated left atrial/pulmonary capillary wedge pressure, tamponade, ventricular arrhythmias, or pneumothorax) requiring ≥14 days of inotropic support or resulting in death within those 14 days. This definition closely aligns with the prior INTERMACS definition of RVF post-LVAD.^15^ Inotropic support was defined as administration of milrinone, adrenaline, isoprenaline, dobutamine, and/or dopamine at any dosage. Temporary RVAD decisions at our institution were based on an integrated assessment including direct assessment of RV function, vasoactive requirements, haemodynamics, and echocardiographic findings, following optimization measures such as pulmonary vasodilator therapy and LVAD flow adjustment.

The full patient cohort (*n* = 203) was divided into 2 groups: those who developed RVF post-LVAD (*n* = 36) and those who did not (*n* = 167). For simplicity, these will be referred to as RVF and non-RVF groups throughout.

### Data collection

Core data were collected prospectively by clinicians and recorded in the Clinical Data Warehouse, a centrally managed institutional database, and these were analyzed retrospectively. Additionally, haemodynamic data were extracted retrospectively from electronic patient records at 2 key timepoints:

1. Preoperative RHC data—from the final RHC performed prior to LVAD surgery.
2. Perioperative pre-CPB pulmonary artery (PA) catheter and arterial-line monitoring data—averaged values between the start of anaesthetic induction and the start of CPB.

Furthermore, the vasoactive-inotropic score (VIS), a measure of cumulative pharmacologic cardiovascular support, was calculated from intraoperative electronic drug chart records as: dopamine (μg/kg/min) + dobutamine (μg/kg/min) + [100 × adrenaline (μg/kg/min)] + [100 × noradrenaline (μg/kg/min)] + [10 × milrinone (μg/kg/min)] +[10,000 × vasopressin (U/kg/min)].^16^ Both mean and maximum VIS values were recorded during this period between anaesthetic induction and the start of CPB.

Parameters from the last preoperative echocardiogram and blood test prior to LVAD surgery were also extracted.

### Statistical analysis

Statistical analyses were performed using STATA version BE/18.5. Missing data were managed appropriately by the statistical software using listwise deletion. Imputation was not performed, as missingness was assumed to be at random. Model comparisons were restricted to the 109 patients with complete data across all candidate variables. Across all analyses, *P* < 0.05 was considered statistically significant.

Data for the RVF and non-RVF groups were compared. Categorical data are presented as numbers and percentages, while continuous variables are presented as mean ± standard deviation if normally distributed, or median with interquartile range (IQR) otherwise. The Shapiro-Wilk test was used to assess normality. Univariate logistic regression was performed for each clinical, echocardiographic, haemodynamic, and blood test parameter to assess associations with RVF post-LVAD.

Least absolute shrinkage-and-selection operator (LASSO) logistic regularization was used for variable selection and coefficient penalization, in order to develop predictive models. All statistically significant variables on univariate analysis and those deemed clinically relevant based on prior literature were entered into this analysis. Two models were generated: (1) Preoperative model—using preoperative variables only, and (2) Pre-CPB model—combining preoperative and perioperative (pre-CPB) markers. Fifteen variables were initially entered into the Preoperative model, and 14 variables were entered into the Pre-CPB model, prior to LASSO selection. These included demographic, echocardiographic, haemodynamic, and biochemical markers. As an external benchmark, the EUROMACS-RHF risk score was calculated for each patient. The predictive performance of each model was assessed using AUC values and receiver operating characteristic (ROC) curves were plotted: (1) Preoperative model, (2) Pre-CPB model, and (3) EUROMACS-RHF risk score. The DeLong test was used to compare differences in predictive performance between the 3 models. Calibration of the Pre-CPB model was assessed using the Hosmer-Lemeshow test, with a *P*-value >.05 indicating acceptable model fit. Internal validation was performed using bootstrap resampling with 1000 replications. In addition, a calibration plot was generated to visually assess agreement between predicted and observed probabilities of RVF post-LVAD.

The optimal classification threshold was identified post-hoc using Youden’s index (sensitivity + specificity − 1). Sensitivity, specificity, positive predictive value (PPV), and negative predictive value (NPV) were calculated at this threshold (**Table S1**). For EUROMACS-RHF, diagnostic performance was assessed at the published high-risk threshold (≥4.5).

## RESULTS

A total of 203 patients who underwent LVAD implantation with magnetically levitated devices (HeartWare or HeartMate III) without concomitant RVAD insertion between 2013 and 2023 were included in the analysis. Early post-implant RVF occurred in 36 patients (17.7%), of whom 13 required RVAD implantation. The average age was 51.0 (40.0-58.0) years (median [IQR]). One hundred and sixty patients (78.8%) were males. One hundred and ninety-eight patients (97.5%) were on a bridge-to-transplant strategy. One hundred and eighty-three patients (90.1%) received a HeartWare device, while the remaining 20 patients (9.85%) received a HeartMate III device. Baseline characteristics of the RVF and non-RVF groups are presented in **Table 1**.

**Table 1.** Baseline Characteristics of Patients Undergoing LVAD Implantation Stratified by RVF Outcome

Variable	RVF (*n* = 36)	Non-RVF (*n* = 167)	Univariate logistic regression		
			Odds ratio	95% CI	*P*-value
Age (years)	**54.0 (49.0-59.0)**	**50.0 (38.0-57.0)**	**1.04**	**1.00-1.07**	**.034**
Gender (*n*, %)			1.26	0.560-2.85	.574
Male	26 (72.2)	128 (76.6)			
Female	10 (27.8)	39 (23.4)			
Ethnicity (*n*, %)					
White	26 (72.2)	117 (70.1)	–	–	–
Black/Black-British	1 (2.78)	20 (12.0)	1.48	0.494-4.40	.486
Asian/Asian-British	4 (11.1)	17 (10.2)	0.787	0.215-2.88	.717
Other	0 (0.00)	2 (1.20)	4.72	0.286-78.0	.278
Body mass index (kg/m^2^)	27.1 ± 4.01	26.8 (23.6-30.1)	0.962	0.897-1.03	.278
Blood group (*n*, %)					
Group O	16 (43.8)	69 (37.2)	–	–	–
Group A	**10 (28.8)**	**60 (35.0)**	**0.361**	**0.143-0.913**	**.031**
Group B	9 (13.7)	25 (17.5)	0.696	0.253-1.92	.484
Group AB	1 (1.37)	5 (3.65)	0.650	0.072-5.89	.702
INTERMACS status (*n*, %)					
Profile 1	3 (8.33)	53 (31.7)	0.879	0.584-1.32	.538
Profile 2	17 (47.2)	76 (45.5)			
Profile 3	14 (38.9)	30 (18.0)			
Profile 4-7	2 (5.56)	7 (4.19)			
Ischaemic heart disease (*n*, %)	16 (44.4)	50 (29.9)	0.888	0.407-1.94	.666
Diabetes (*n*, %)	6 (16.7)	28 (16.8)	1.23	0.490-3.10	.655
Previous myocardial infarction (*n*, %)	17 (47.2)	48 (28.7)	1.16	0.533-2.54	.703
Preoperative IV inotrope therapy (*n*, %)	33 (91.7)	118 (70.7)	2.78	0.926-8.33	.068
Previous intra-aortic balloon pump (*n*, %)	6 (16.7)	29 (17.4)	1.22	0.482-3.09	.674
Implantable cardioverter-defibrillator at implant (*n*, %)	22 (61.1)	91 (54.5)	1.31	0.605-2.83	.494
Previous sternotomy (*n*, %)	7 (19.4)	22 (13.2)	0.509	0.143-1.80	.295
Concomitant surgery (*n*, %)	5 (13.9)	7 (4.19)	0.918	0.192-4.38	.914

Categorical data reported as *n* (percentage). Continuous data reported as mean ± standard deviation if normally distributed, or median with interquartile range (IQR) if non-normally distributed. Bolded values indicate statistically significant results (*P* < 0.05).

Older age (odds ratio [OR]: 1.04 per year, *P* = 0.034) was significantly associated with the development of RVF post-LVAD (**Table 1**). Other variables such as gender, comorbidities, and clinical characteristics did not differ significantly between RVF and non-RVF patients (**Table 1**). Preoperative RHC parameters were compared between the RVF and non-RVF groups (**Table 2**). The median interval between the RHC and LVAD surgery was 21 days (IQR: 13-40). Overall, preoperative RHC parameters were similar between the 2 groups. The median right atrial pressure to pulmonary capillary wedge pressure ratio (RAP:PCWP) ratio was higher in the RVF group than the non-RVF group (0.540 [0.370-0.677] vs 0.453 [0.333-0.594]). The RAP:PCWP ratio had the numerically largest effect size (OR: 4.59), but this still did not reach the threshold of statistical significance (*P* = 0.084) on univariate analysis (**Table 2**). Perioperative haemodynamic markers derived from PA catheter and arterial-line monitoring averaged over the time period between anaesthetic induction and the start of CPB are summarized in **Table 3**. Patients who developed RVF had significantly lower pulmonary pressures during this period, including PA systolic pressure (OR: 0.963, *P* = 0.011), PA diastolic pressure (OR: 0.945, *P* = 0.017), and PA mean pressure (OR: 0.948, *P* = 0.008) (**Table 3**). Preoperative echocardiographic markers and blood test values were also compared between the RVF and non-RVF groups (**Tables S2 and S3**).

**Table 2.** Preoperative Right Heart Catheterization Parameters in RVF vs Non-RVF Groups

Variable	RVF (*n* = 36)	Non-RVF (*n* = 167)	Univariate logistic regression		
			Odds ratio	95% CI	*P*-value
Aortic oxygen saturation (%)	98.0 (97.0-99.0)	97.5 (95.5-99.0)	1.19	0.942-1.50	.146
Pulmonary oxygen saturation (%)	59.1 ± 7.74	54.6 ± 10.4	1.01	0.964-1.05	.757
Right atrial mean pressure (mmHg)	10.4 ± 4.90	12.0 (8.00-15.0)	1.02	0.952-1.10	.544
RV systolic pressure (mmHg)	51.7 ± 15.7	54.0 (40.0-65.0)	0.985	0.958-1.01	.270
RV diastolic pressure (mmHg)	7.03 ± 3.90	8.00 (4.00-10.0)	0.948	0.862-1.04	.276
RV end-diastolic pressure (mmHg)	11.9 ± 6.26	14.5 ± 5.47	0.997	0.924-1.08	.932
PA systolic pressure (mmHg)	53.7 ± 18.4	54.6 ± 16.7	0.986	0.961-1.01	.269
PA diastolic pressure (mmHg)	27.9 ± 7.78	27.6 ± 8.51	0.981	0.933-1.03	.458
PA mean pressure (mmHg)	27.3 ± 10.7	33.1 ± 10.7	0.977	0.939-1.02	.261
Pulmonary capillary wedge pressure (mmHg)	23.4 ± 7.13	25.0 ± 7.46	0.974	0.920-1.03	.375
Transpulmonary gradient (mmHg)	16.0 (10.0-19.0)	12.0 (8.00-16.0)	0.973	0.910-1.04	.411
Cardiac output (L/min)	3.88 (3.14-4.51)	3.07 (2.58-3.77)	0.844	0.559-1.27	.420
Cardiac index (L/min/m^2^)	2.11 (1.64-2.36)	1.65 (1.38-1.88)	1.05	0.507-2.18	.894
Pulmonary vascular resistance (wood units)	4.22 ± 2.67	3.80 (2.73-5.32)	0.960	(0.807-1.14	.642
Pulmonary artery pulsatility index	2.45 (1.56-4.22)	2.29 (1.36-3.82)	0.979	0.865-1.11	.733
Right atrial pressure to pulmonary capillary wedge pressure ratio	0.429 (0.344-0.588)	0.494 ± 0.245	4.59	0.815-25.9	.084

Data reported as mean ± standard deviation if normally distributed, or median with interquartile range (IQR) if non-normally distributed.

Abbreviations: CI, confidence interval; PA, pulmonary artery; RV, right ventricle; RVF, right ventricular failure.

**Table 3.** Intraoperative Pre-CPB Haemodynamic Variables in RVF vs Non-RVF Groups

Variable	RVF (*n* = 36)	Non-RVF (*n *= 167)	Univariate logistic regression		
			Odds ratio	95% CI	*P*-value
Central venous pressure (mmHg)	16.2 ± 7.10	15.0 ± 5.55	1.04	0.971-1.10	.285
PA systolic pressure (mmHg)	**37.4 ± 14.8**	**44.9 ± 14.4**	**0.963**	**0.935-0.991**	**.011**
PA diastolic pressure (mmHg)	**20.9 ± 9.76**	**25.0 (19.0-31.0)**	**0.945**	**0.902-0.990**	**.017**
PA mean pressure (mmHg)	**27.3 ± 10.7**	**33.0 (26.0-40.0)**	**0.948**	**0.911-0.986**	**.008**
Mean arterial pressure (mmHg)	72.7 ± 9.71	73.0 (68.0-79.0)	0.967	0.918-1.02	.204
Heart Rate (beats/min)	88.0 ± 15.4	89.3 ± 19.0	0.996	0.975-1.02	.700
Peripheral oxygen saturation (%)	98.0 (97.0-99.0)	98.0 (97.0-99.0)	1.01	0.870-1.17	.918
Pulmonary artery pulsatility index	0.808 (0.593-1.54)	1.42 (0.877-1.96)	0.973	0.749-1.26	.835
Mean arterial pressure to central venous pressure ratio	4.76 (3.53-6.33)	4.91 (4.01-6.80)	1.01	0.928-1.10	.818
Vasoactive-inotropic score					
Average	3.24 (2.18-6.32)	2.50 (1.65-4.53)	1.06	0.970-1.16	.189
Max	5.50 (2.75-9.50)	4.00 (2.50-8.00)	1.05	0.982-1.13	.146

Values averaged between anaesthetic induction and start of cardiopulmonary bypass, stratified by RVF versus non-RVF groups. Data presented as mean ± standard deviation if normally distributed, or median with interquartile range (IQR) if non-normally distributed. Bold values indicate statistically significant results (*P* < 0.05).

Abbreviations: CI, confidence interval; PA, pulmonary artery; RVF, right ventricular failure.

Model development was performed in the analytic cohort with complete data (*n* = 109). Baseline characteristics were broadly similar between included and excluded patients, except device distribution (**Table S4**). Least absolute shrinkage-and-selection operator regularization was used to develop a Preoperative model incorporating preoperative variables (demographics, clinical variables, echocardiography, and RHC) only. This retained 4 predictors (age, severe RV dysfunction on echocardiography, moderate-severe tricuspid regurgitation, and RAP:PCWP ratio) (**Table 4**). The second LASSO-derived model was the Pre-CPB model, where perioperative (pre-CPB) markers from PA catheter and arterial line monitoring were included in addition to preoperative markers. This initially retained 7 predictors. Collinearity was assessed among retained variables, which demonstrated a strong positive correlation (r = 0.867) between average VIS and max VIS, while all other variables demonstrated no collinearity (r = −0.2 to +0.3) (**Table S5**). To minimize redundancy, average VIS was excluded, as max VIS demonstrated a stronger association with RVF. This led to a final model consisting 6 predictors (age, severe RV dysfunction on echocardiography, RAP:PCWP ratio, PAPi, MAP, and max VIS) (**Table 5**).

**Table 4.** LASSO-derived Preoperative Prediction Model

Variable	Odds ratio	95% CI	*P*-value
Age (years)	1.04	0.998-1.10	.060
Severe RV dysfunction (on echocardiography)	4.71	1.64-13.6	.004
Moderate-severe tricuspid regurgitation	1.81	0.679-4.80	.236
Right atrial pressure to pulmonary capillary wedge pressure ratio	2.71	0.363-20.3	.331

Back-transformed regression coefficients (β) and corresponding odds interpretation for each predictor retained in the LASSO-derived Preoperative model. Abbreviations: CI, confidence interval; RV, right ventricle.

**Table 5.** LASSO-derived Pre-CPB Prediction Model

Variable	Odds ratio	95% CI	*P*-value
Age (years)	1.04	0.982-1.10	.176
Severe RV dysfunction (on echocardiography)	6.52	1.73-24.6	.006
Right atrial pressure to pulmonary capillary wedge pressure ratio	4.53	0.389-52,7	.228
Pulmonary artery pulsatility index Pre-CPB	1.37	0.940-1.99	.101
Mean arterial pressure Pre-CPB (mmHg)	0.877	0.798-0.965	.007
Max vasoactive-inotropic score Pre-CPB	1.12	0.998-1.27	.053

Back-transformed regression coefficients (β) and corresponding odds interpretation for each predictor of RVF post-LVAD retained in the LASSO-derived Pre-CPB model, which incorporated both preoperative and Pre-CPB intraoperative markers.Abbreviations: CI, confidence interval; CPB, cardiopulmonary bypass; PA, pulmonary artery.

**Figure 1.**
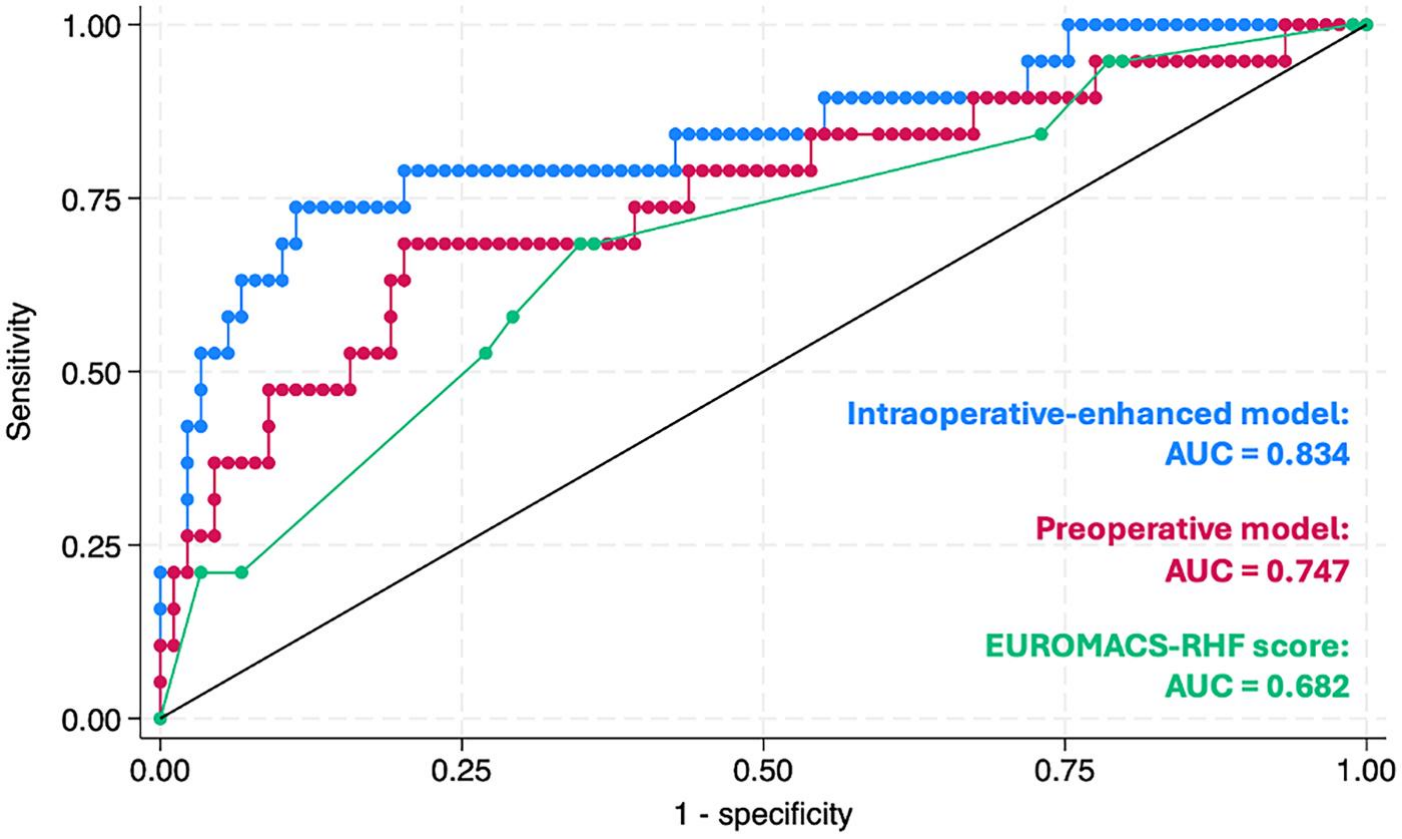
Receiver Operating Characteristic Curves Comparing Predictive Models for RVF Post-LVAD. ROC curves for (1) Preoperative model (developed in this study), (2) Pre-CPB model (developed in this study), and (3) EUROMACS-RHF risk score (applied to our patient cohort). Abbreviation: AUC, area under the curve

As an external benchmark, the predictive performance of the EUROMACS-RHF risk score was evaluated within our cohort. Twenty-two (16.2%) patients were in the low-risk category, 8 patients (5.88%) were in the intermediate-risk category, and 106 patients (77.9%) were in the high-risk category. Our Preoperative model achieved an AUC of 0.747 (95% confidence interval [CI]: 0.610—0.884), demonstrating moderate discrimination (**Figure 1**). Our Pre-CPB model demonstrated strong predictive performance, achieving an AUC of 0.834 (0.717—0.951) (**Figure 1**). The EUROMACS-RHF score yielded an AUC of 0.682 (0.547—0.817) in our cohort, indicating similar performance to the derivation cohort (AUC: 0.70) (**Figure 1**). The Pre-CPB model significantly outperformed both the EUROMACS-RHF score (DeLong test, *P* = 0.0299) and our Preoperative model (DeLong test, *P* = 0.049). There was no significant difference in predictive performance between the Preoperative model developed in our study and the EUROMACS-RHF score (DeLong test, *P* = 0.222).

In a sensitivity analysis restricted to HeartWare LVAD patients, the Pre-CPB model demonstrated similar predictive performance (AUC: 0.857) to the primary analysis. Calibration of the Pre-CPB model was acceptable (Hosmer-Lemeshow test, *P* = .252) (**Figure S1**). Bootstrap internal validation (1000 replications) demonstrated consistent model performance, with an AUC of 0.81 (95% CI 0.70—0.92), based on 943 successful replications. The probability that an LVAD patient will develop RVF, according to our Pre-CPB model, can be calculated as shown in **Figure S2**. The optimal risk cutoff to define high-risk was identified from Youden’s index as ≥25%, with sensitivity of 74%, specificity of 89%, PPV of 54%, and NPV of 94%. For comparison, at the EUROMACS-RHF high-risk threshold (≥4.5), PPV was 21% and NPV was 89% in our cohort, compared to PPV of 43% and NPV of 84% calculated from the original EUROMACS risk score derivation cohort.^7^

## DISCUSSION

This study has three key findings. First, no individual preoperative haemodynamic variable derived from right heart catheter (RHC) alone independently predicted RVF post-LVAD. Second, a multivariable model combining preoperative RHC, echocardiography, demographics, and biochemical markers, achieved only moderate discrimination. Third, incorporating perioperative haemodynamic markers prior to the start of CPB demonstrated significantly improved predictive performance compared to preoperative predictions, including the EUROMACS-RHF risk score, which is a widely validated risk tool.

Our results emphasize the limitations of relying solely on preoperative markers.^1^^,^^5^ Right heart catheterization plays a central role in LVAD work-up, offering assessments of RV preload, afterload, and contractility.^17^ However, these measurements are obtained under stable, closed-pericardium conditions in minimally stressed patients, and may not capture dynamic changes in RV reserve under anaesthesia, altered preload and afterload, and peri-induction haemodynamic shifts.^10^ In contrast, perioperative measurements in our study were obtained between anaesthetic induction and initiation of CPB, a phase reflecting the RV’s dynamic response to physiological stress rather than baseline haemodynamic status. In our cohort, no individual RHC-derived parameter was significant on univariate analysis. While some studies report similarly limited predictive value, others have identified markers such as RA: PCWP to be significant.^18^^,^^19^ However, these findings are inconsistently replicated, underscoring the limited reliability of preoperative haemodynamics as standalone predictors. Although RAP:PCWP ratio approached significance and was retained in multivariable modelling, this Preoperative model showed only moderate predictive value (AUC: 0.73), comparable to established preoperative scores such as EUROMACS-RHF, RVFRS, and Drakos scores, which typically report AUCs of ∼0.70-0.74.^7–9^

In contrast, haemodynamic assessment including VIS, MAP, and PAPi during the perioperative period between induction and initiation of CPB (ie, prior to LVAD implantation and before any device-related haemodynamic support) showed significant incremental value (AUC: 0.83). These parameters capture haemodynamic responses to anaesthetic induction and pre-CPB optimization, reflecting dynamic RV loading conditions that are not apparent preoperatively.^10^ Although pre-CPB PAPi was not significant in univariate analysis, it was retained following penalized variable selection and contributed to overall model performance in combination with other predictors, consistent with its established physiological relevance in assessing RV function. Our findings support the concept that haemodynamic stress can unmask subclinical RV dysfunction. Kang et al^20^ demonstrated greater predictive value of preoperative PAPi in patients receiving inotropic support, suggesting that haemodynamic stress reveals latent RV dysfunction. Similarly, Cacioli et al^21^ demonstrated improved predictive accuracy of PAPi following pharmacological vasodilator challenge during RHC. Our findings extend these insights to the perioperative setting, where endogenous and iatrogenic stressors provide a real-time physiological “stress test” of RV reserve.

Moreover, our results complement a recent study by Gudejko et al,^13^ who showed that haemodynamic parameters recorded after chest closure—particularly CVP and PAPi—were predictive of RVF. We demonstrate that measurements obtained even earlier in the perioperative course—prior to CPB initiation—already provide a meaningful predictive signal. This period is characterized by rapid shifts in preload, systemic vascular resistance, and myocardial contractility induced by anaesthesia and early surgical manipulation, which may unmask limited RV reserve not evident preoperatively.^10^ This offers a critical temporal advantage, allowing earlier risk stratification before major surgical and device-related haemodynamic alterations, and greater opportunity for proactive intraoperative planning.

Importantly, our Pre-CPB model outperformed the EUROMACS-RHF score, which yielded an AUC of 0.68 in our cohort—closely mirroring its original derivation value (AUC: 0.70).^7^ This external benchmarking supports the generalizability of our cohort. Furthermore, the lack of significant difference between our Preoperative model and EUROMACS-RHF (*P* = .222) highlights the comparability of preoperative-only approaches. The significant improvement with the Pre-CPB model (*P* = .0299) underscores the value of capturing physiological changes during the early intraoperative window. Overall, our study addresses a key unmet clinical need: the lack of structured tools to support intraoperative decision-making.^6^ Existing scores are preoperative and offer limited real-time guidance once in the operating theatre.^6^ Decisions regarding RVAD initiation, inotrope escalation, or delayed chest closure are often made without objective thresholds.^6^ Our Pre-CPB model, based on routinely monitored variables, offers a pragmatic solution. Nonetheless, risk stratification tools cannot replace the clinical judgement of an experienced cardiac surgeon and anaesthetist on a case-by-case basis, but may guide in-theatre decision-making. Prospective validation and development of further real-time intraoperative risk calculators are required to enable earlier, more confident intervention before irreversible RV decompensation occurs.

### Limitations

This study has several limitations. It was a single-centre, retrospective study, with a relatively modest sample size, which may limit external validity, as the findings may reflect local centre-specific practices. However, the similar performance of EUROMACS-RHF in our cohort when compared to the original multicentre derivation cohort suggests good generalizability. Nonetheless, larger multicentre studies and prospective validation are needed. Second, intraoperative echocardiography data were unavailable retrospectively. Future studies should assess whether combining intraoperative echocardiography with haemodynamics further enhances predictive accuracy.^22^ Third, comparisons with other risk scores (eg, RVFRS, Drakos) were not possible due to missing variables. Finally, this model does not guide the preoperative choice between transplantation versus LVAD, which remains based on preoperative haemodynamics and echocardiography and the decision of the multidisciplinary team prior to surgery.

## CONCLUSION

This study demonstrates that perioperative haemodynamic markers prior to CPB offer significantly greater accuracy for predicting RVF post-LVAD, compared to preoperative RHC, echocardiographic, and old scoring systems. Routine real-time data from the operating room (Pre-CPB model) provide a more accurate means of identifying high-risk patients while there is still an opportunity to intervene early. These findings support a shift towards real-time, perioperative risk stratification strategies to inform decisions such as RVAD initiation and optimization of inotropic support. Prospective validation and integration into intraoperative decision-support tools will be essential for translating these insights into improved clinical outcomes.

## Supplementary Material

ivag215_Supplementary_Data

## Data Availability

The data used in this study are available on request.
