# Supplementary material for: Haemodynamics from Induction to Cardiopulmonary Bypass Improve Prediction of Right Ventricular Failure Following Left Ventricular Assist Device
Source: Interdiscip Cardiovasc Thorac Surg. 2026 Jul 29;41(8):ivag215. doi: 10.1093/icvts/ivag215 (PMC13480432; doi:10.1093/icvts/ivag215)
Supplement: Supplementary file 1 [file ivag215_supplementary_data.docx]

**Table S1**

|  | **Formula** |
| --- | --- |
| Positive predictive value (PPV) | $\frac{Sensitivity x Prevalence}{\left( Sensitivity x Prevalence \right)+[\left( 1-Specificity \right)x \left( 1-Prevalence \right)]}$ |
| Negative predictive value (NPV) | $\frac{Specificity x (1-Prevalence)}{\left[ Specificity x \left( 1-Prevalence \right) \right]+[\left( 1-Sensitivity \right)x Prevalence]}$ |

**Table S2 – Preoperative echocardiographic parameters in RVF vs non-RVF groups**

| **Variable** | **RVF (n = 36)** | **Non-RVF**  **(n = 167)** | **Univariate Logistic Regression** | | |
| --- | --- | --- | --- | --- | --- |
|  |  |  | **Odds ratio** | **95% CI** | **p-value** |
| IVSd (cm) | 1.00 (0.880 – 1.12) | 0.900 (0.800 – 1.00) | 3.78 | 0.912 – 15.6 | 0.067 |
| LVIDd (cm) | 6.65 ± 1.13 | 6.79 ± 1.06 | 0.887 | 0.632 – 1.25 | 0.490 |
| LVIDs (cm) | 6.17 ± 1.20 | 6.03 ± 1.14 | 1.11 | 0.804 – 1.53 | 0.531 |
| LVPWd (cm) | 0.877 ± 0.232 | 0.810 (0.700 – 1.00) | 0.832 | 0.344 – 2.01 | 0.683 |
| TAPSE (cm) | 1.35 (1.20 – 1.60) | 1.33 ± 0.423 | 2.22 | 0.950 – 5.20 | 0.066 |
| EF (%) | 20.7 ± 7.24 | 20.0 (14.0 – 26.8) | 0.999 | 0.957 – 1.04 | 0.952 |
| LV EDV (ml) | 216 (130 – 284) | 214 (165 – 281) | 1.00 | 0.997 – 1.00 | 0.687 |
| LV ESV (ml) | 161 (125 – 232) | 179 (127 – 238) | 1.00 | 0.997 – 1.00 | 0.726 |
| **RA size**  **Severe dilation (n, %)**  **Moderate-severe dilation (n, %)** | **17 (47.2)**  **20 (55.6)** | **27 (16.2)**  **42 (25.1)** | **3.88**  **3.05** | **1.77 – 8.51**  **1.42 – 6.53** | **0.001**  **0.004** |
| **LA size**  **Severe dilation (n, %)**  **Moderate-severe dilation (n, %)** | **26 (72.2)**  **30 (83.3)** | **62 (37.1)**  **74 (44.3)** | **4.67**  **7.80** | **1.98 – 11.0**  **2.62 – 23.2** | **<0.001**  **<0.001** |
| RV size  Severe dilation (n, %)  Moderate-severe dilation (n, %) | 4 (11.1)  6 (16.7) | 10 (5.99)  15 (8.98) | 1.91  1.97 | 0.564 – 6.48  0.708 – 5.50 | 0.298  0.194 |
| **LV size**  Severe dilation (n, %)  **Moderate-severe dilation (n, %)** | 23 (63.9)  **28 (77.8)** | 75 (44.9)  **97 (58.1)** | 2.05  **2.35** | 0.972 – 4.33  **1.01 – 5.47** | 0.059  **0.048** |
| RV function  Severe impairment (n, %)  Moderate-severe impairment (n, %) | 7 (19.4)  13 (36.1) | 30 (18.0)  46 (27.5) | 1.10  1.49 | 0.439 – 2.76  0.693 – 3.21 | 0.839  0.307 |
| LV function  Severe impairment (n, %)  Moderate-severe impairment (n, %) | 36 (100)  36 (100) | 149 (89.2)  157 (94.0) | N/A  N/A | N/A  N/A | N/A  N/A |
| Tricuspid regurgitation  Severe regurgitation (n, %)  Moderate-severe regurgitation (n, %) | 5 (13.9)  10 (27.8) | 23 (13.8)  68 (40.7) | 0.982  0.537 | 0.346 – 2.78  0.243 – 1.19 | 0.972  0.125 |
| Mitral regurgitation  Severe regurgitation (n, %)  Moderate-severe regurgitation (n, %) | 17 (47.2)  25 (69.4) | 55 (32.9)  99 (59.3) | 1.77  1.49 | 0.854 – 3.68  0.687 – 3.24 | 0.124  0.311 |
| Pulmonary regurgitation  Severe regurgitation (n, %)  Moderate-severe regurgitation (n, %) | 0 (0.00)  2 (1.37) | 0 (0.00)  7 (4.19) | N/A  1.13 | N/A  0.223 – 5.68 | N/A  0.887 |

This table presents echocardiographic parameters from the last preoperative echocardiogram in the RVF and non-RVF groups. Non-parametric data are presented as numbers and their corresponding percentages. Continuous data are presented as mean ± standard deviation if normally distributed, or median with interquartile range (IQR) for non-normally distributed data. Results of univariate logistic regression to identify variables that are significantly associated (p<0.05) with RVF following LVAD are presented. Their odds ratios, 95% confidence intervals and p-values are provided. Statistically significant results are bolded. N/A refers to variables where perfect prediction prevented these values from being obtained.

*IVSd = interventricular septal thickness at end-diastole; LVIDd = left ventricular internal dimension in diastole; LVIDs = left ventricular internal dimension in systole; LVPWd = left ventricular posterior wall thickness at end-diastole; LVEDV = left ventricular end-diastolic volume; LVESV = left ventricular end-systolic volume; TAPSE = tricuspid annular plane systolic excursion; EF = ejection fraction; RA = right atrium; LA = left atrium; RV = right ventricle; LV = left ventricle*

**Table S3 – Preoperative blood test parameters in RVF vs non-RVF groups**

| **Variable** | **RVF (n = 36)** | **Non-RVF**  **(n = 167)** | **Univariate Logistic Regression** | | |
| --- | --- | --- | --- | --- | --- |
|  |  |  | **Odds ratio** | **95% CI** | **p-value** |
| Hb (g/L) | 131 ± 19.6 | 128 (115 – 141) | 1.01 | 0.992 – 1.03 | 0.244 |
| RBC (x10^12^/L) | 4.38 ± 0.651 | 4.36 ± 0.676 | 1.05 | 0.611 – 1.80 | 0.861 |
| Platelets (x10^9^/L) | 185 (157 – 247) | 201 (157 – 256) | 0.998 | 0.994 – 1.00 | 0.433 |
| WBC (x10^9^/L) | 7.70 (6.35 – 10.2) | 8.20 (6.80 – 10.8) | 0.981 | 0.896 – 1.07 | 0.686 |
| Neutrophils (x10^9^/L) | 5.20 (3.95 – 7.60) | 5.90 (4.60 – 8.00) | 1.00 | 0.914 – 1.10 | 0.975 |
| Lymphocytes (x10^9^/L) | 1.28 ± 0.465 | 1.40 (1.00 – 1.90) | 0.551 | 0.292 – 1.04 | 0.066 |
| Sodium (mmol/L) | 135 ± 5.54 | 135 ± 4.93 | 1.01 | 0.939 – 1.08 | 0.805 |
| Potassium (mmol/L) | 4.29 ± 0.517 | 4.20 (3.80 – 4.50) | 1.28 | 0.681 – 2.40 | 0.444 |
| Urea (mmol/L) | 7.35 (5.95 – 10.8) | 8.80 (6.00 – 11.7) | 0.983 | 0.901 – 1.07 | 0.706 |
| Creatinine (mmol/L) | 87.5 (70.0 – 120) | 103 (75.0 – 126) | 0.997 | 0.988 – 1.01 | 0.470 |
| GFR (ml/min) | 55.9 ± 21.3 | 56.7 ± 16.4 | 0.997 | 0.970 – 1.03 | 0.843 |
| Bilirubin (μmol/min) | 22.0 (12.5 – 45.0) | 19.0 (13.0 – 26.0) | 1.02 | 1.00 – 1.03 | 0.052 |
| ALT (IU/L) | 24.0 (18.0 – 32.0) | 31.0 (18.0 – 53.0) | 1.00 | 0.998 – 1.00 | 0.869 |
| CRP (mg/L) | 19.0 (4.00 – 91.0) | 12.0 (4.00 – 47.0) | 1.00 | 0.999 – 1.01 | 0.104 |
| BNP (ng/L) | 1289 ± 795 | 918 (560 – 1730) | 1.00 | 1.00 – 1.00 | 0.797 |
| Troponin (ng/L) | 61.0 (34.6 – 308) | 105 (36.5 – 454) | 1.00 | 1.00 – 1.00 | 0.551 |

This table presents the results of routine preoperative blood tests in the RVF and non-RVF groups. Data are presented as mean ± standard deviation if normally distributed, or median with interquartile range (IQR) for non-normally distributed data. Results of univariate logistic regression to identify variables that are significantly associated (p<0.05) with RVF following LVAD are presented. Their odds ratios, 95% confidence intervals and p-values are provided. Statistically significant results are bolded.

*Hb = haemoglobin; RBC = red blood cell count; WBC = white blood cell count; GFR = glomerular filtration rate; ALT = alanine aminotransferase; CRP = C-reactive protein; BNP = brain natriuretic peptide*

**Table S4 – Comparison of included versus excluded patients in final modelling**

| **Variable** | **Included (n=109)** | **Excluded (n = 94)** | **p-value** |
| --- | --- | --- | --- |
| Age (years) | 52.0 (44.0 – 58.0) | 49.5 (36.0 – 57.0) | 0.171 |
| Female gender (n, %) | 23 (21.1) | 26 (27.7) | 0.277 |
| White ethnicity (n, %) | 78 (76.5) | 65 (76.5) | 1.00 |
| **Device Type**  **HeartWare (n, %)**  **HeartMate 3 (n, %)** | **91 (83.5)**  **18 (16.5)** | **92 (97.9)**  **2 (2.1)** | **0.0006** |
| Previous IV inotrope therapy (n, %) | 78 (72.2) | 73 (81.1) | 0.194 |
| Previous IABP use (n, %) | 13 (13.0) | 22 (25.0) | 0.055 |
| Concomitant surgery (n, %) | 6 (5.50) | 6 (6.45) | 0.777 |

**Table S5 – Correlation between predictor variables**

|  | **Age** | **Severe RV dysfunction** | **Moderate-severe TR** | **RAP:PCWP** | **PAPi** | **MAP** | **VIS (max)** |
| --- | --- | --- | --- | --- | --- | --- | --- |
| **Age** | – |  |  |  |  |  |  |
| **Severe RV dysfunction** | -0.108 | – |  |  |  |  |  |
| **Moderate-severe TR** | 0.114 | 0.155 | – |  |  |  |  |
| **RAP:PCWP** | -0.110 | 0.187 | 0.253 | – |  |  |  |
| **PAPi** | 0.155 | -0.060 | 0.007 | -0.149 | – |  |  |
| **MAP** | 0.073 | 0.002 | -0.194 | -0.042 | -0.033 | – |  |
| **VIS (max)** | 0.040 | 0.134 | 0.048 | -0.130 | -0.139 | -0.139 | – |
| **VIS (average)** | -0.032 | 0.191 | 0.064 | -0.072 | -0.123 | -0.117 | **0.867** |

This table presents the results of routine preoperative blood tests in the RVF and non-RVF groups. Data are presented as mean ± standard deviation if normally distributed, or median with interquartile range (IQR) for non-normally distributed data. Results of univariate logistic regression to identify variables that are significantly associated (p<0.05) with RVF following LVAD are presented. Their odds ratios, 95% confidence intervals and p-values are provided. Statistically significant results are bolded.

*RV = right ventricle; TR = tricuspid regurgitation; RAP:PCWP = right atrial pressure to pulmonary capillary wedge pressure ratio; PAPi = pulmonary artery pulsatility index, MAP = mean arterial pressure; VIS = vasoactive inotrope score; max = maximum*

**Figure S1 – Calibration Plot for the Intraoperative-enhanced Model**

This figure presents a calibration plot comparing predicted and observed probabilities for RVF post-LVAD. The dashed diagonal line (reference line) represents perfect calibration. Green circles indicate observed event rates across predicted risk bins, with 95% confidence intervals shown. All 95% confidence intervals cross the reference line, indicating acceptable calibration. The rug plot along the x-axis illustrates the distribution of individual predicted probabilities. Calibration metrics are shown in the top-left corner of the plot. *O:E = observed-to-expected ratio; CITL = calibration-in-the-large; AUC = area under the receiver operating characteristic curve*

**Figure S2 – Formula to calculate the predicted risk of RVF for a patient using our Intraoperative-enhanced model**

This formula can be used to calculate the probability of RVF post-LVAD based on our intraoperative-enhanced model. All variables are entered in the original measurement units shown. Binary variables should be entered as 0 (absent) or 1 (present). The result is the predicted probability of RVF for an individual patient.
